# Effects of Glucose Load and Nateglinide Intervention on Endothelial Function and Oxidative Stress

**DOI:** 10.1155/2013/849295

**Published:** 2013-04-07

**Authors:** Leilei Wang, Lixin Guo, Lina Zhang, Yan Zhou, Qinghua He, Zheng Zhang, Meng Wang

**Affiliations:** ^1^VIP Department, Beijing Hospital of the Ministry of Health, Dongdan Dahua, Road Number One, Beijing 100730, China; ^2^Endocrinology and Metabolism Department, Beijing Hospital of the Ministry of Health, Dongdan Dahua, Road Number One, Beijing 100730, China; ^3^Ultrasound Division, Beijing Hospital of the Ministry of Health, Dongdan Dahua, Road Number One, Beijing 100730, China; ^4^Laboratory Division, Beijing Hospital of the Ministry of Health, Dongdan Dahua, Road Number One, Beijing 100730, China

## Abstract

We analysed endothelial function and oxidative stress in patients with abnormal glucose metabolism, the effect of glucose load, and the impact of nateglinide. 109 participants were grouped into newly diagnosed diabetes, prediabetes, and control. Fasting plasma glucose (FPG), postprandial plasma glucose (PPG), glycosylated haemoglobin (HbA_1c_), and glycated albumin (GA) varied significantly among the study groups (*P* < 0.01). Nitric oxide (NO) and insulin resistance index (HOMA-IRI) levels were markedly different between the newly diagnosed diabetes and the control (*P* < 0.01). Glucose loading lowered flow-mediated endothelium-dependent dilation (FMEDD), NO, and superoxide dismutase (SOD) (*P* < 0.01). Fasting and glucose loading FMEDD, FPG, PPG, HbA_1c_, and GA were negatively correlated (*r* = −0.4573, −0.4602, −0.3895, −0.3897, and *r* = −0.4594, −0.4803, −0.4494, −0.3885; *P* < 0.01), whereas NO, SOD, and HOMA-**β** were positively correlated (*r* = 0.2983, 0.3211, 0.311, and *r* = 0.1954, 0.361, 0.2569; *P* < 0.05). After the treatment with nateglinide, significant decreases in FPG, PPG, GA, HbA1C, endothelin-1(ET-1), malondialdehyde (MDA), and HOMA-IRI were observed, whereas FMEDD, NO, and SOD increased (*P* < 0.01). Thus, the study demonstrated the adverse effect of glucose load on endothelial function and oxidative stress. Nateglinide lowers blood glucose, reduces insulin resistance and oxidative stress, and improves endothelial function in newly diagnosed diabetes.

## 1. Introduction

The incidence of diabetes is increasing at an alarming rate worldwide. The prevalence of diabetes, reported as about 346 million by WHO, is predicted to advance further in the future if not treated [1]. Diabetes, a chronic metabolic disorder characterised by hyperglycaemia and glucose intolerance, is associated with both macrovascular and microvascular complications [2]. Diabetes-induced vascular complications such as cardiovascular, cerebrovascular, and peripheral vascular diseases adversely affect the life expectancy and quality of life of the patients.

At the cellular level, endothelial and vascular smooth cell dysfunction along with abnormal coagulation system is associated with the pathogenesis of diabetes-linked vascular complications [3]. Among these factors, vascular endothelial dysfunction is a key pathological factor in promoting diabetic vascular complications. Oxidative stress, one of the major mechanisms responsible for vascular endothelial dysfunction, is mediated through increased reactive oxygen species (ROS). Elevated ROS is generated through diverse biochemical pathways impacted by hyperglycaemic conditions [4]. In normal vascular endothelium, regulatory mediators, such as NO, prostanoids, endothelin-1(ET-1), and angiotensin II, ensure vascular homeostasis [5]. However, ROS scavenges NO, thus limiting its availability for vascular homeostasis [6]. Compromised endothelial regeneration and angiogenesis processes ultimately lead to diabetes-associated micro- and macrovascular complications [7]. 

While studying the effect of glucose fluctuations on oxidative stress in patients with diabetes, it was observed that there was an increased triggering of oxidative stress under conditions of glucose fluctuations or glucose swings when compared with chronic sustained hyperglycaemia [8]. Glycaemic variability is being related to the occurrence as well as the increase in severity of coronary artery disease (CAD) in type 2 diabetic patients [9]. Fluctuations in FPG within the normal limits in nondiabetic healthy individuals are associated with aggravated arterial stiffness [10]. However, Major-Pedersen et al. (2008) reported that oral glucose load does not cause endothelial dysfunction in healthy individuals when the mean glucose level is at 5.6 mmol/L and insulin is at 27.2 mmol/L, at 2 hours after glucose [11]. Nevertheless, a recent study has shown that oral glucose load attenuated flow-mediated dilation in brachial artery of healthy individuals [12].

Progression of type 2 diabetes is characterised by loss of early insulin secretion, leading to postprandial hyperglycaemia. This ultimately leads to insulin resistance [5]. Usage of an insulin secretagogue can selectively enhance early meal-induced insulin secretion, resulting in improved postprandial hyperglycaemia, thus preventing type 2 diabetes. Nateglinide, an insulin secretagogue, has been shown to selectively enhance early meal-induced insulin secretion, leading to meal time glucose control. An earlier study demonstrated that nateglinide can effectively normalise the glycaemic response curve after an oral glucose load in patients with impaired glucose tolerance (IGT) [13].

In the light of these earlier observations, the objective of the present study was to detect vascular endothelial function and oxidative stress levels in patients with abnormal glucose metabolism and to understand the changes after the glucose load. The study also aimed at analysing the impact of nateglinide on vascular endothelial function and oxidative stress in newly diagnosed patients with diabetes.

## 2. Patients and Methods

### 2.1. Study Population and Study Design

The study protocol was approved by the Ministry of Health Beijing Hospital Ethics Committee. Informed consent was obtained from all the participants.

The study participants were recruited from Beijing Hospital Internal Medicine, Endocrinology Clinic from June to December of 2010. Inclusion criteria were no previous history of (1) diabetes or impaired glucose regulation (2) hypertension, and (3) dyslipidemia. Subjects were aged between 35 and 65 years (average age of 52.62 ± 8.51 years), of which 49 were male and 60 were female. All subjects were unrelated and belonged to Han Dynasty. Exclusion criteria were (1) diabetes with acute complications such as diabetic ketoacidosis, diabetic lactic acidosis, hyperosmolar nonketotic hyperglycaemic coma and hypoglycaemia syndrome, (2) secondary diabetes, (3) hypertension, coronary heart disease, dyslipidaemia, vascular inflammation, vascular stenosis and atherosclerotic peripheral vascular lesions, (4) patients with abnormal renal function (serum creatinine > 178 *μ*mol/L, blood urea nitrogen > 9 mmol/L) and abnormal liver function (transaminases greater than twice the normal), (5) serious autoimmune and blood diseases, (6) presence of fever, infectious diseases as well as dementia and other mental illnesses, (7) current use of antihypertensive drugs, lipid lowering drugs and other drug, which affect endothelial function, (8) patients who refused treatment and/or comprehensive review after 4 weeks. 109 participants in the recruitment met the above the criteria. All participants underwent oral glucose tolerance tests (OGTT), conducted flow-mediated endothelium-dependent dilation (FMEDD), and detected other indicators.

#### 2.1.1. Data Collection

Patients' demographics, vital statistics (height, weight, body mass index (BMI), blood pressure) and medical history were collected from all participants. Fasting plasma glucose (FPG), postprandial plasma glucose (PPG), glycosylated haemoglobin (HbA_1c_), glycated albumin (GA), total cholesterol (TC), triglyceride (TG), low-density lipoprotein cholesterol (LDL-C), high-density lipoprotein cholesterol (HDL-C), creatinine (CRE), alanine aminotransferase (ALT), aspartate aminotransferase (AST), uric acid (UA), serum insulin (INS), serum C-peptide nitric oxide (NO), endothelin-1(ET-1), malondialdehyde (MDA), and superoxide dismutase (SOD) were detected in all participants.

#### 2.1.2. Diagnostic Criteria for Diabetes

Diagnosis of diabetes was based on the WHO specified diagnostic criteria (2006). On the basis of the results of the oral glucose tolerance test (OGTT), patients were diagnosed to have either diabetes (FPG ≥ 7.0 mmol/L or 2 h plasma glucose (PG) ≥ 11.1 mmol/L), IGT (2 h PG 7.8–11.0 mmol/L), or impaired fasting glucose (IFG) regulation (OGTT FPG 6.1–6.9 mmol/L) [14]. Normal glucose metabolism was defined by OGTT values 6.0 mmol/L or less (FPG) and 7.7 mmol/or less (2 h PG). On the basis of OGTT results, 109 participants were separated into 3 groups, namely, newly diagnosed type 2 diabetes group (*n* = 43; 25 males and 18 females with mean age of 53.40 ± 8.99 years), prediabetes group (*n* = 33; 12 males and 21 females with mean age of 52.88 ± 9.20 years), and control group (*n* = 33; 12 males and 21 females with mean age of 51.36 ± 7.15 years).

Among the newly diagnosed diabetic patients, 32 (19 males, 13 females, mean age of 54.94 ± 7.41 years) patients who signed informed consent were given nateglinide (120 mg tid), 3 patients were provided diet management and exercise, 3 were administered intensive insulin therapy, 3 other patients were on other antidiabetic drug therapy and 2 patients were lost to follow-up. In the newly diagnosed type 2 diabetes group, medication compliance was 98.10%.

Only the 32 diabetic patients treated with nateglinide had a repeat of all measurements, and the weight of the 32 diabetic patients was measured after 4 weeks of treatment.

#### 2.1.3. Treatment Protocol

32 diabetic patients were treated with nateglinide at a dosage of 120 mg tid. Analyses regarding the changes of endothelial function and oxidative stress in these patients were compared pre- and posttherapy after 4 weeks of treatment interval.

### 2.2. Laboratory Methods

The various biochemical tests performed included FPG, PPG, total cholesterol (TC), triglyceride (TG), low-density lipoprotein cholesterol (LDL-C), high-density lipoprotein cholesterol (HDL-C), creatinine (CRE), alanine aminotransferase (ALT), aspartate aminotranferase (AST), uric acid (UA), serum insulin (INS), serum C-peptide, and glycosylated haemoglobin (HbA1c). These tests were carried out in biochemical laboratory. Determination of glycated albumin (GA) was performed using a test kit provided by the Japanese Asahi Kasei company.

Other biochemical tests included NO estimation by nitrate reductase method, MDA by thiobarbituric acid (TBA) method, total superoxide dismutase (SOD) by xanthine oxidase method, and ET-1 by enzyme linked immunosorbent assay (test kit from Nanjing Jiancheng Bioengineering Institute and the American R and B Company; kit sensitivity: 0.5 *μ*g/L).

### 2.3. Flow-Mediated Endothelium-Dependent Dilation (FMEDD)

FMEDD was performed using Philips (Italy) Philips IU22 high-resolution multifunction colour Doppler ultrasonic diagnostic apparatus with a probe frequency of 4–8 MHz. Subjects were asked to rest comfortably in supine position at least 10 min before the test, with the right arm above the elbow for imaging the brachial artery 2–15 cm above the antecubital crease [15]. The longitudinal section image was taken by adjusting the probing depth and gaining recognition to measure the brachial artery diameter as *D0* (simultaneous to electrocardiographic *R* wave). 

### 2.4. Reactive Hyperaemia Test

Blood pressure cuff was tied on the forearm and was inflated to 280 mmHg for 5 min. Reactive hyperaemia results by deflation during which brachial artery image is recorded after 50 s to 60 s and at 90 s, as brachial artery diameter (*D1*). Reactive hyperaemia (in percentage) is calculated as brachial artery diameter after expansion as follows:

(1) $$\begin{array}{c} \text{FMEDD}=\frac{\left(D1-D0\right)}{D0}\times 100\%\\ \left(\text{normal reference values}\;\left(11\pm 2\right)\%\right), \end{array}$$

see [16].

During the entire testing process, the ultrasonic probe was used at a fixed location by the same ultrasound professional who used the same testing equipment to operate. The persons measuring the above measurements did not know which group the patients belonged to.

### 2.5. Insulin Sensitivity and *β*-Cell Function Evaluation

HOMA of insulin sensitivity and basal *β*-cell function are calculated as

(2) $$\begin{array}{c} \text{HOMA-IRI}=\frac{\text{FPG}\times \text{fasting insulin (Fins)}}{22.5}, \end{array}$$

see [17]. Assume

(3) $$\begin{array}{c} \text{HOMA}\;\beta \text{-cell}\;\text{function}\left(\text{HOMA-}\beta \right)=\frac{20\times \text{Fins}}{\left(\text{FPG}-3.5\right)}. \end{array}$$

#### 2.5.1. Glucose Load

Various tests were measured both while fasting and 2 hours after the consumption of 75 g oral glucose.

### 2.6. Statistical Analysis

Data entry was performed using epidata3.0 and double parallel entry. All data were analysed by SAS9.1.2 software. Results of continuous variables were presented as mean ± standard deviation. For comparisons between the 3 groups, analysis of variance (ANOVA) was used in case of normal distribution and homogeneity of variance between groups. Nonparametric test methods were used for incomplete variance and in case of not meeting the normal distribution. Multiple comparison methods were used to check if the differences between the two groups were statistically significant. Repetitive measure ANOVA was used for comparison between fasting and 2 hours after glucose load. To compare pre- and post-treatments of newly diagnosed type 2 diabetes, the single sample *t*-test was used if the difference met the normal distribution; signed rank test was used if the difference did not meet the normal distribution. Pearson's and Spearman's correlation analyses were used to examine correlations between the various indicators. Multiple comparison methods were used to check statistical significance in differences between the two groups. For all analyses, *P* value of less than .05 was considered to be statistically significant.

## 3. Results

### 3.1. Analysis of Clinical Metabolic Parameters in Different Glucose Metabolism Status and after Glucose Load

BMI, liver, and kidney functions were comparable among prediabetes, newly diagnosed type 2 diabetes, and control groups (*P* > .05). Although ALT and AST values were non-significantly elevated in newly diagnosed type 2 diabetes groups when compared with the control group, uric acid level was significantly increased in the former group. (*P* < .05) (Table 1).

Statistically significant differences were observed in FBG, PPG, DPG, HbA_1c_, and GA among the control group, prediabetes group, and newly diagnosed type 2 diabetes group (*P* < .001). However, increase in total cholesterol, TG, and LDL-C was not significantly different in these 3 groups (*P* > .05). HDL-C and HOMA-IRI were significantly different between newly diagnosed type 2 diabetes group and control group (*P* < .05) (Table 2).

Both at fasting and at 2 hours after glucose load, FMEDD and SOD showed a significant difference among the prediabetes, newly diagnosed type 2 diabetes, and control groups (*P* < .01). NO was significantly lowered in both prediabetic and newly diagnosed type 2 diabetes group at fasting and 2 hours after glucose load, when compared with control group (*P* < .01). In addition, fasting plasma NO was significantly decreased in newly diagnosed type 2 diabetes when compared with prediabetes group (*P* < .01). Among the three groups, FMEDD, NO, and SOD levels were significantly decreased after the glucose load when compared with fasting level (*P* < .01). However, the levels of MDA and ET-1 at fasting and 2 hours after glucose load did not differ significantly between the 3 groups. (*P* > .05) In the control group and the newly diagnosed type 2 diabetes group, ET-1 and MDA increased significantly (*P* < .01) (Table 3). Additional data on correlation analysis is provided in Online Resource 1.

The effect of nateglinide treatment on vascular endothelial function and oxidative stress in patients with newly diagnosed type 2 diabetes was also analysed. FPG, PPG, DPG, HbA1C, GA, and HOMA-IRI were significantly decreased after nateglinide therapy (*P* < .001); LDL-C also decreased significantly after nateglinide treatment (*P* < .05). Significant decrease in the levels of ALT and AST were also recorded after treatment (*P* < .05). The decrease in TC, TG, and CRE and the increase in HDL-C and HOMA-*β* after treatment were not significant (*P* > .05) (Table 4).

Treatment with nateglinide significantly increased FMED, NO, and SOD levels both at fasting and 2 hours after glucose load in patients newly diagnosed with type 2 diabetes, (*P* < .001) However, ET-1 and MDA decreased significantly after treatment (*P* < .001). After the treatment, the average weight had no significant difference comparing with the previous (*P* > .05) (Table 5).

## 4. Discussion

### 4.1. Clinical Metabolic Parameters under Different Glucose Metabolism

In obese and overweight population, impairment of endothelial function appears even before the signs of metabolic disorders and cardiovascular diseases are evident [18]. A dose-response relationship between BMI and FMEDD has been demonstrated [19]. However, in the present study, BMI was observed to be not significantly different among the study groups. Although FMEDD decreased with increase in BMI, the difference was not statistically significant (*P* > .05). Thus, the present study did not demonstrate the influence of BMI on endothelial function among the prediabetic, type 2 diabetic, and normal individuals. Oxidative stress and endothelial dysfunctions are observed to increase with high uric acid concentration [20]. Our study too demonstrated significant increase in the levels of uric acid in patients with diabetes compared with the control population. FMEDD was also significantly decreased in patients with diabetes. In addition, uric acid showed a significant negative correlation with fasting SOD and SOD after glucose load. These results suggest a role for uric acid in oxidative stress in patients with diabetes. People with dyslipidaemia, hypertension, and so forth were excluded in this study, it will lead to a somewhat selected population of patients with T2DM and does not represent the majority of the population of diabetic patients who often have high blood pressure as well as hyperlipidaemia. Because hypertension, coronary heart disease, dyslipidaemia, vascular inflammation, vascular stenosis, and atherosclerotic peripheral vascular lesions could affect the vascular endothelial function, the patients with dyslipidaemia, hypertension, and so forth were excluded in order to avoid confounding factors. 

Significant differences in the levels of plasma glucose (FPG, PPG, DPG), HbA1C, and GA among the study groups demonstrate that these parameters reflect the state of glucose metabolism in an individual. Dyslipidaemia is one of the main factors responsible for endothelial dysfunction [21]. Increase in TG is associated with increased free fatty acid (FFA) production resulting in rise in mitochondrial ROS production. Moreover, reduction of intracellular GSH antioxidant defence system with increase in FFA further increases oxidative stress [22]. However, since in this study, patients with abnormal blood lipid profile were excluded, increase in the levels of TC, TG, and LDL-C, although not significant, suggests that endothelial function and oxidative stress are influenced by dyslipidaemia.

### 4.2. Glucose Load on Oxidative Stress and Endothelial Function

Many clinical ultrasound data have shown that, for early diabetes, FMEDD was impaired both while fasting as well as after glucose load but nitroglycerin-mediated endothelium-independent dilation (NMEID) had no significant differences when compared with normal subjects [23, 24]. The fact that nonendothelium-dependent vascular dilation function and vascular smooth muscle cell functions are normal in early diabetic people suggests that endothelial dysfunction precedes vascular structural changes during the progress of diabetic vascular disease in chronic diabetes. In this study, FMEDD (fasting and 2 hours after the glucose load) was negatively correlated with FPG, PPG, HbA1C, GA, and positively correlated with NO, SOD, and HOMA-*β*. This suggests that blood glucose levels can impact endothelial dysfunction. At the same time, a significant correlation between FMEDD, NO, and SOD indicates the strong effect of NO and antioxidative stress on endothelium-dependent vasodilation. The negative correlation between FMEDD (2 hours after glucose load) and LDL-C may be due to increased oxidative stress associated with the acute blood glucose fluctuations. This is supported by earlier findings that increased oxidative stress is triggered in diabetic patients under conditions of glucose fluctuations or glucose swings when compared with chronic sustained hyperglycaemia [8]. Moreover, oxidation of LDL-C to form Ox-LDL-C, can also increase superoxide anions (O_2_^−^), leading to endothelial cell dysfunction [25].

Many studies have indicated that endothelial dysfunction is present in prediabetes groups, including IGT or IFG [26–28]. In this study, FMEDD, NO, and SOD during fasting and 2 hours after glucose load were significantly different among the study groups. Several studies have shown that HOMA-IRI is significantly increased and HOMA-*β* significantly reduced in patients with diabetes [29, 30]. Results of our study are consistent with these observations. In this study HOMA-IRI and BMI, FPG, PPG, HbA1C showed significant positive correlations whereas HOMA-*β* and FPG, HbA1C, and GA were negatively correlated. This suggests that with high blood glucose levels, *β*-cell function was reduced and insulin resistance was more apparent as oxidative stress can aggravate pancreatic *β* cells dysfunction by promoting early apoptosis of *β* cells. In addition, HOMA-*β* and FMEDD (fasting and 2 hours after glucose load) were positively correlated. With reduced *β*-cell function, the NO synthesis may be inhibited, which damages the endothelial NO synthase (eNOS), leading to endothelial dysfunction [31].

Acute blood glucose fluctuations after glucose load can trigger a series of adverse reactions, including increased insulin resistance, oxidative stress, and endothelial dysfunction. Endothelial cells in a stable high glucose environment have a certain degree of adaptive capacity, whereas the blood glucose fluctuations impaired endothelial ability to adapt [32]. In a prospective follow up study in type 2 diabetic patients, postprandial hyperglycaemia was suggested as an independent risk factor for cardiovascular disease [33]. Number of animal experiments confirmed that acute glucose fluctuations increased adhesion of monocytes to vascular endothelium, which had more impact leading to endothelial dysfunction compared with changes due to persistent hyperglycaemia [34, 35]. Glucose fluctuations after glucose load can quickly inhibit brachial artery endothelium-dependent vasodilation in patients with impaired glucose tolerance and diabetes [36–38]. Our study results are also in agreement with these observations, as FMEDD, NO, and SOD after glucose load were significantly decreased compared with that while fasting. In contrast, ET-1 and MDA were significantly higher among newly diagnosed diabetes group. At the same time, a positive correlation was seen for DPG and FPG, PPG, HbA1C, GA, HOMA-IRI, and TG. Both fasting and 2 hours glucose load DPG and FMEDD, NO, SOD were negatively correlated. Thus, the vulnerable endothelial function in diabetes is damaged further after glucose fluctuations.

### 4.3. Effect of Nateglinide on Endothelial Function and Oxidative Stress

Many antioxidant drugs are being used to reduce the toxicity of high glucose by acting against ROS and related pathways to reduce oxidative stress. In this study, we evaluated the effect of nateglinide therapy on endothelial function and oxidative stress in patients with newly diagnosed type 2 diabetes. Nateglinide is believed to reduce glucose autoxidation and hence reduce the generation of oxygen free radicals in the polyol pathway and in the glycolytic pathway by lowering blood sugar [39]. In the study by Major-Pedersen et al. (2008), nateglinide could improve endothelial function by reducing postprandial hyperglycaemia and stimulating early phase insulin secretion in patients with insulin resistance [11]. Nateglinide reduces oxidative stress and restores carotid artery intima-media thickness by strict control of blood glucose in patients with type 2 diabetes [40]. In this study, the average weight had no significant difference comparing with the previous after the treatment. Therefore, an impact on endothelial function and oxidative stress of the change in weight was firstly excluded. In this part, FPG, PPG, DPG, HbA1C, GA, HOMA-IRI, ET-1, MDA were significantly decreased in posttreatment condition with nateglinide when compared with pretreatment. FMEDD, NO, and SOD during fasting and 2 hours after glucose load were significantly higher. LDL-C, ALT, and AST levels were also significantly reduced after treatment with nateglinide. Although TC and TG decreased and HDL-C and HOMA-*β* increased after nateglinide treatment, the changes were statistically nonsignificant (*P* > .05). 

On the basis of these findings, we conclude that nateglinide can significantly lower blood glucose, reduce insulin resistance, improve endothelial function, and reduce oxidative stress. The mechanism involved in nateglinide-mediated improvement of endothelial function and oxidative stress may be as follows: (1) by acting on pancreatic *β* cells, nateglinide may rapidly and briefly inhibit adenosine triphosphate (ATP) sensitive potassium channel (K_ATP_ channel) to remodel early phase insulin secretion, which has a similar physiological pattern of insulin secretion. Early phase secretion of insulin can act directly on the liver and inhibit hepatic glucose output to reduce postprandial blood glucose levels, which can reduce the effect of blood glucose fluctuation on endothelial function and oxidative stress; (2) nateglinide can inhibit lipolysis and reduce free fatty acid levels and oxidative stress; (3) early phase insulin secretion can maintain normal endothelial function, thus inhibiting the secretion of inflammatory factors.

Hence, reducing the blood glucose fluctuations to mitigate endothelial dysfunction and oxidative stress is beneficial as it delays the development of diabetic vascular complications.

## Figures and Tables

**Table 1:** Analysis of the general clinical data.

Group	Control group	Prediabetes groups	Newly diagnosed type 2 diabetes group	*F*/*χ* ^2^ value	*P* value
Number of patients (M/F)	33 (12/21)	33 (12/21)	43 (25/18)		
Age (years)	51.36 ± 7.15	52.88 ± 9.20	53.40 ± 8.99	1.78^a^	.411
BMI	24.76 ± 3.60	25.75 ± 3.13	25.69 ± 2.93	2.55^a^	.280
ALT	25.58 ± 6.84	26.91 ± 12.00	31.49 ± 14.21	3.30^a^	.192
AST	26.03 ± 5.64	26.12 ± 7.55	28.74 ± 10.35	1.07^a^	.586
CRE	65.30 ± 10.44	66.19 ± 13.96	65.60 ± 13.32	0.04	.959
UA	302.21 ± 73.79	324.12 ± 74.42	348.60 ± 84.44	3.31	.040*

M/F: male/female; BMI: body mass index; ALT: alanine aminotransferase; AST: aspartate aminotransferase; CRE: creatinine; UA: uric acid.

^a^Nonparametric analysis method was used.

*Significant between newly diagnosed type 2 diabetes and control groups.

**Table 2:** Analysis of relative biochemical indicators.

Group	Control group	Prediabetes groups	Newly diagnosed type 2 diabetes group	*F*/*χ* ^2^ value	*P* value
Number of patients (M/F)	33 (12/21)	33 (12/21)	43 (25/18)		
FPG	5.43 ± 0.45	6.08 ± 0.53	7.54 ± 1.17	72.87^a^	<.001*
PPG	6.49 ± 0.89	8.80 ± 1.09	14.55 ± 3.19	90.78^a^	<.001*
DPG	1.19 ± 0.59	2.72 ± 1.31	7.01 ± 2.45	78.85^a^	<.001*
HbA_1_C (%)	5.96 ± 0.31	6.50 ± 0.01	7.40 ± 0.97	67.02^a^	<.001*
GA	12.73 ± 1.00	13.71 ± 0.93	16.59 ± 1.81	75.93^a^	<.001*
TC	5.48 ± 0.55	5.52 ± 0.97	5.84 ± 1.74	2.44^a^	.296
TG	1.51 ± 0.49	1.80 ± 0.90	2.16 ± 1.17	5.78^a^	.056
LDL-C	3.07 ± 0.61	3.14 ± 0.75	3.23 ± 0.71	0.48	.618
HDL-C	1.36 ± 0.39	1.24 ± 0.26	1.17 ± 0.26	6.27^a^	.043**
HOMA-IRI	1.85 ± 1.43	2.69 ± 2.15	3.48 ± 2.74	7.68^a^	.022**
HOMA-*β*	80.49 ± 59.30	81.45 ± 66.45	52.99 ± 41.58	5.35^a^	.069

FPG: fasting plasma glucose; PPG: postprandial plasma glucose; DPG: diphosphoglycerate plasma glucose; HbA_1_C: glycosylated haemoglobin; GA: glycated albumin; TC: total cholesterol; TG: triglyceride; LDL-C: low-density lipoprotein cholesterol; HDL-C: high-density lipoprotein cholesterol; HOMA-IRI: homeostasis model method of assessment-insulin resistance index; HOMA-*β*: homeostasis model method of assessment *β*-cell function.

^a^Nonparametric analysis method used.

*Significant among the 3 groups.

**Significant between newly diagnosed type 2 diabetes and control group.

**Table 3:** Differences between FMEDD, NO, ET-1, MDA, after SOD after glucose load.

Group		Control group	Prediabetes groups	Newly diagnosed type 2 diabetes group	*F*/*χ* ^2^ value	*P* value
Number of patients (M/F)		33 (12/21)	33 (12/21)	43 (25/18)		
FMEDD	Fasting	17.79 ± 3.91	15.91 ± 4.40	12.16 ± 2.93	28.81^a^	<.001*
	After glucose load	14.07 ± 3.15	11.99 ± 4.37	9.42 ± 3.18	16.09	<.001*
	*P* value	<.001	<.001	<.001		
NO	Fasting	174.76 ± 67.25	153.69 ± 61.67	128.02 ± 52.91	12.28^a^	.002***
	After glucose load	153.63 ± 60.06	138.13 ± 60.79	117.90 ± 48.25	9.43^a^	.009**
	*P* value	<.001	.0006	<.001		
ET-1	Fasting	1.44 ± 1.51	1.62 ± 1.29	1.93 ± 1.79	1.87^a^	.392
	After glucose load	1.66 ± 1.54	1.54 ± 1.47	2.13 ± 1.86	2.17^a^	.338
	*P* value	<.001	.3878	.0046		
MDA	Fasting	5.41 ± 1.32	5.72 ± 1.18	5.76 ± 1.29	0.8	.454
	After glucose load	6.03 ± 1.62	6.14 ± 1.64	6.31 ± 1.65	0.49^a^	.783
	*P* value	<.001	.1938	.0003		
SOD	Fasting	169.24 ± 22.35	143.94 ± 17.37	118.51 ± 17.26	63.73^a^	<.001*
	After glucose load	159.85 ± 22.57	127.88 ± 14.24	100.93 ± 17.37	77.17^a^	<.001*
	*P* value	.0052	.0011	.0002		

FMEDD: flow-mediated endothelium-dependent dilation; NO: nitric oxide; ET-1: endothelin-1; MDA: malondialdehyde; SOD: superoxide dismutase.

^a^Using nonparametric analysis method.

*Significant among the 3 groups.

**Significantly different in newly diagnosed type 2 diabetes and control groups.

***Significantly different in newly diagnosed type 2 diabetes and control groups, prediabetes groups, and newly diagnosed type 2 diabetes group.

**Table 4:** Comparison of biochemical parameters in newly diagnosed type 2 diabetes before and after treatment with nateglinide.

Group	Before taking nateglinide	After taking nateglinide	Difference in levels before and after nateglinide treatment	*P* values
Number of patients (M/F)	32 (19/13)	32 (19/13)		
Weight	71.35 ± 10.47	70.81 ± 9.52	0.54 ± 4.13	.805
FPG	7.44 ± 0.69	6.74 ± 0.52	0.69 ± 0.49	<.001*
PPG	14.16 ± 2.53	10.11 ± 2.09	4.05 ± 2.43	<.001
DPG	6.72 ± 2.28	3.36 ± 2.07	3.36 ± 2.34	<.001
HbA_1_C (%)	7.26 ± 0.58	6.73 ± 0.35	0.53 ± 0.50	<.001*
GA (%)	16.43 ± 1.03	14.39 ± 0.64	2.04 ± 0.81	<.001
TC	5.54 ± 0.81	5.22 ± 0.84	0.32 ± 0.76	.062*
TG	2.10 ± 1.07	1.81 ± 0.58	0.30 ± 0.87	.063*
LDL-C	3.17 ± 0.71	2.84 ± 0.60	0.33 ± 0.55	.002
HDL-C	1.18 ± 0.26	1.22 ± 0.20	0.04 ± 0.25	.252*
HOMA-IRI	3.62 ± 2.91	2.86 ± 2.18	0.76 ± 1.75	.008
HOMA-*β*	56.69 ± 45.04	59.86 ± 44.71	3.17 ± 25.03	.072
ALT	31.63 ± 14.67	28.31 ± 11.19	3.31 ± 7.73	.044*
AST	28.97 ± 10.80	26.66 ± 8.10	2.31 ± 4.61	.003*
CRE	66.75 ± 13.31	68.22 ± 12.79	1.47 ± 6.27	.451*
UA	353.56 ± 92.01	358.28 ± 76.40	4.72 ± 62.86	.869*

FPG: fasting plasma glucose; PPG: postprandial plasma glucose; DPG: diphosphoglycerate plasma glucose; HbA_1_C: glycosylated haemoglobin; GA: glycated albumin; TC: total cholesterol; TG: triglyceride; LDL-C: low-density lipoprotein cholesterol; HDL-C: high-density lipoprotein cholesterol; HOMA-IRI: Homeostasis model method of assessment (HOMA) insulin resistance index; HOMA-*β*: HOMA *β*-cell function; ALT: alanine aminotransferase; AST: aspartate aminotransferase; CRE: creatinine; UA: uric acid.

*Using nonparametric analysis method.

**Table 5:** Changes in FMEDD, NO, ET-1, MDA, and SOD in newly diagnosed type 2 diabetes after treatment with nateglinide.

Group		Before taking nateglinide	After taking nateglinide	Difference in levels before and after nateglinide treatment	*P* values
Number of patients (M/F)		32 (19/13)	32 (19/13)		
FMEDD	Fasting	12.20 ± 3.21	16.06 ± 4.23	3.86 ± 4.11	<.001
	After glucose load	9.4 ± 3.43	12.81 ± 4.01	3.41 ± 4.50	.0002
NO	Fasting	134.15 ± 58.55	173.64 ± 72.44	39.49 ± 35.22	<.001*
	After glucose load	123.76 ± 53.62	145.18 ± 62.97	20.52 ± 50.96	<0.001*
ET-1	Fasting	2.16 ± 1.81	1.40 ± 1.23	0.75 ± 0.92	<.001*
	After glucose load	2.31 ± 1.88	1.85 ± 1.56	0.46 ± 0.73	<.001*
MDA	Fasting	5.64 ± 1.22	4.66 ± 1.01	0.98 ± 0.77	<.001*
	After glucose load	6.19 ± 1.59	5.39 ± 1.03	0.80 ± 0.95	<.001*
SOD	Fasting	118.53 ± 17.30	146.81 ± 21.02	28.81 ± 15.48	<.001
	After glucose load	99.84 ± 17.09	125.28 ± 16.58	25.44 ± 13.00	.0002

FMEDD: flow-mediated endothelium-dependent dilation; NO: nitric oxide; ET-1: endothelin-1; MDA: malondialdehyde; SOD: superoxide dismutase.

*Using nonparametric analysis method.
